# Botanical defence: medicinal plants as botanical insecticides against *Locustana pardalina* Walker (Orthoptera: Acrididae)

**DOI:** 10.3389/finsc.2026.1833553

**Published:** 2026-07-30

**Authors:** Afika-Amazizi N. Mbuyiswa, Hlalanathi Y. Gwanya, Ompelege J. Phokwe, Nomagugu Gxaba, Madira C. Manganyi

**Affiliations:** 1 Department of Nature Conservation, Mangosuthu University of Technology, Durban, South Africa; 2 Botany Section, Department of Biological and Environmental Sciences, Walter Sisulu University, Mthatha, Eastern Cape, South Africa; 3 Department of Biology and Environmental Science, Sefako Makgatho Health Sciences University, Pretoria, South Africa

**Keywords:** botanical insecticides, food security, *L. pardalina*, metabolites, outbreaks, pest management

## Abstract

**Background:**

Food crop infestations caused by the insect pest *Locustana pardalina* threaten global food security, particularly in developing regions. This pest can devastate entire crops, exacerbating malnutrition and economic instability. Traditionally, *L. pardalina* outbreaks have been managed using synthetic chemical insecticides, which, while effective, pose significant environmental risks by harming nontarget species and contaminating ecosystems. Given these concerns, there is an increasing demand for environmentally friendly alternatives. Medicinal plant extracts, known for their biodegradability, cost-effectiveness, and active metabolites, offer promising solutions.

**Method:**

A systematic literature search was conducted across Google Scholar, PubMed, ScienceDirect, and SpringerLink using keywords related to *Locustana pardalina*, food security, outbreaks, botanical insecticides, metabolites, and pest management. The search (1988–2024) conducted used predefined eligibility criteria and identified 120 relevant studies on the biological control of various insects spanning approximately five to eight crop systems.

**Results:**

These plant-based insecticides have demonstrated the ability to repel various insect pests, including *L. pardalina*, and could provide an effective method for controlling locust swarms while mitigating the harmful effects of chemical pesticides. This review discusses the potential of medicinal plant extracts as sustainable alternatives for managing *L. pardalina* infestations, highlighting the active compounds that show promise in pest control and their benefits over conventional chemical insecticides.

**Conclusion:**

In conclusion, medicinal plant extracts represent a sustainable and environmentally friendly alternative to synthetic insecticides for managing *Locustana pardalina* infestations. Their biodegradability, cost-effectiveness, and bioactive metabolites offer effective pest control while minimising ecological harm, highlighting their potential to support food security and reduce the negative impacts associated with chemical pesticides.

## 1 Introduction

**Figure 1:**
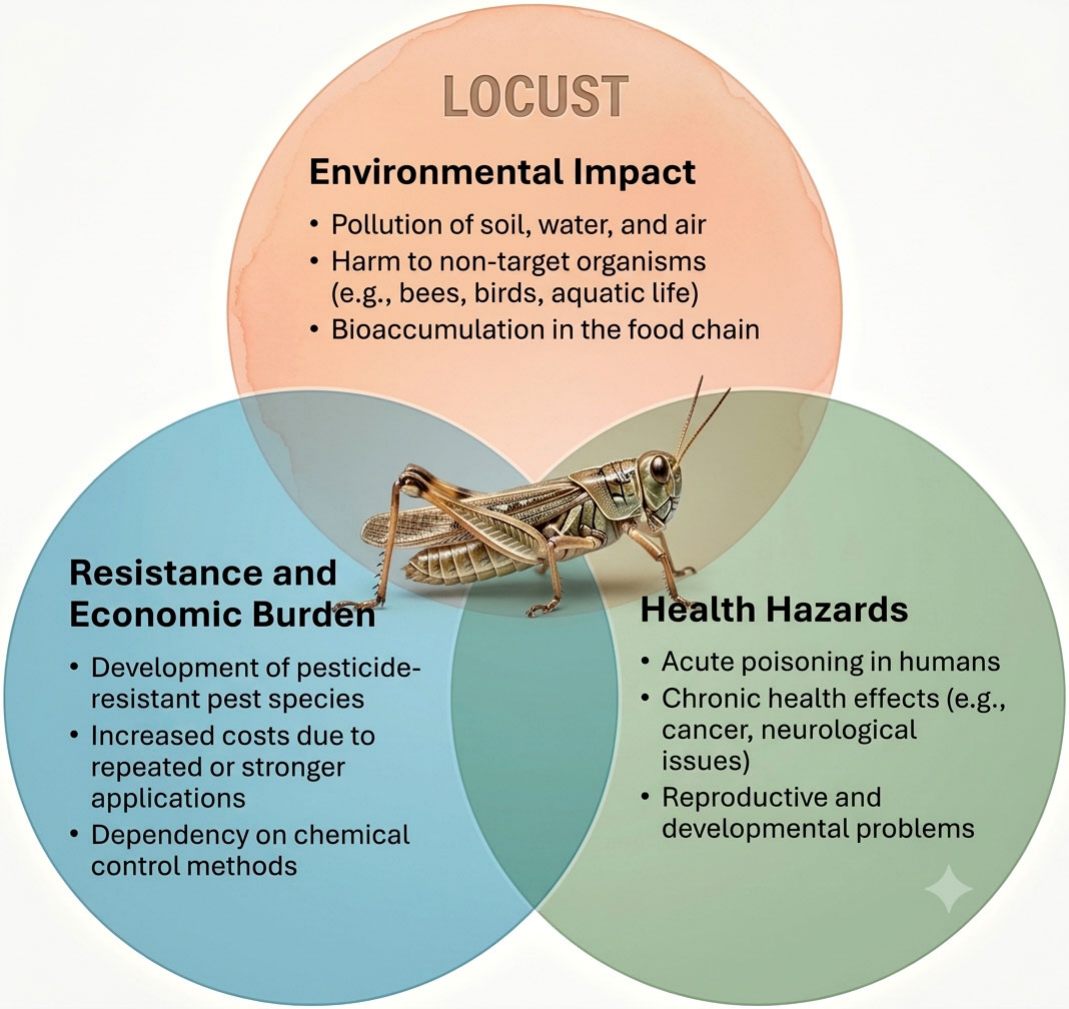
Conceptual overview of phytochemical approaches for controlling brown locust, highlighting the major classes of plant-derived insecticidal compounds and their associated prospects and challenges.

Invasive insect pests, which have spread across more than 50% of the African continent, pose a serious threat to food security, particularly in regions that rely on subsistence agriculture. The brown locust, *Locustana pardalina* (Walk.) (Orthoptera: Acrididae), is one of the major crop pests that can cause significant damage to crops and pastures, particularly, but not restricted to, Southern Africa (1). These locusts, infamous for their capacity to form massive swarms that destroy pastureland and crops (1), can proliferate quickly in conducive environments, resulting in outbreaks that cover large areas. During such outbreaks, swarms of *L. pardalina* can decimate wheat, sorghum, and other staple crops, causing significant losses to the agricultural sector. In addition to harming food crops, grazing areas are also impacted, which lowers livestock feed availability and jeopardises food security (2, 3). In regions that depend on agriculture for subsistence and commercial farming, locust plagues disrupt livelihoods, aggravate poverty, and place immense strain on national economies. Locust outbreaks can have a significant negative impact on the environment, including yield losses, decreased nutrient composition, higher control costs, and long-term effects on land recovery and soil fertility (4). Locust outbreaks that have affected food security, rural economies, and the livelihoods of millions of people worldwide, especially in areas heavily reliant on subsistence farming, have been documented in a body of research (5–9). Although strides have been made in managing outbreaks, currently employed control strategies for locust swarms primarily involve the use of synthetic chemical insecticides. These insecticides, which are frequently sprayed aerially, have been shown to be successful in lowering locust populations and minimising crop damage (10). Nonetheless, there are several drawbacks to this strong reliance on chemical pesticides. The main issue is that over time, locust populations may develop resistance to pesticides, which reduces the effectiveness of these chemicals. Furthermore, there are significant environmental dangers associated with the widespread use of synthetic pesticides, such as water body contamination and harm to nontarget species (6), including pollinators. Moreover, misuse of these compounds can result in bioaccumulation, which disturbs nearby ecosystems and reduces biodiversity. Chemical control methods also impact human health, especially for farm workers and rural communities who may be exposed to pesticide residues or drift. The high expense of synthetic pesticides makes their application even more challenging, particularly in locations with limited resources where timely access to these chemicals is sometimes problematic (10). As a result, afflicted areas are more susceptible to prolonged locust infestations. Considering these difficulties, an increasing number of researchers are looking for environmentally friendly and sustainable locust management methods. The application of botanical insecticides as biopesticides is one potential strategy (11). Moreover, botanical insecticides containing numerous bioactive substances, including flavonoids, terpenoids, alkaloids, limonoids, essential oils, and other secondary metabolites, have shown effects on related locusts (8, 12–15). Pesticides derived from plants provide a number of benefits, including higher biodegradability, environmental safety, and a lower risk of harming nontarget species compared with the use of traditional chemical insecticides (16). Furthermore, the complex diversity of active chemicals with numerous modes of action decreases the likelihood of resistance development in insect pests such as locusts. Nonetheless, laboratory and field research on the use of medicinal herbs specifically against *L. pardalina* is still lacking; most studies are extrapolated from related locusts and lepidopteran or hemipteran species (15). The effectiveness of plant-derived pesticides in controlling swarms of brown locusts should be prioritised, even if they have been researched for other insect pests (8). Hence, the purpose of this review is to investigate the potential of using botanical insecticides to manage *L. pardalina*. This review synthesises historical data, evaluates contemporary chemical management limitations, and explores the biochemical potential of indigenous plant-derived secondary metabolites as sustainable, environmentally congruent protection against *L. pardalina* infestations (13–15) (**Figure 1**).

## 2 Materials and methods

### 2.1 Study design and literature search strategy

A comprehensive literature search was carried out using platforms such as Google Scholar, PubMed, ScienceDirect, and SpringerLink. Keywords including “*L. pardalina*, food security, outbreaks, botanical insecticides, metabolites, and pest management” were used to identify relevant peer-reviewed sources. To support language refinement and improve the clarity of the manuscript, generative AI tools were employed in a limited and supervised manner. SciSpace Copilot (Version 1.62) and Google Gemini (Version 1.5 Pro) were used to assist in summarising key points and restructuring selected content. All AI-assisted outputs were critically reviewed, fact-checked, and revised by the authors to ensure scientific accuracy, originality, and compliance with ethical standards.

## 3 The biology and feeding behaviour of *L. pardalina*

### 3.1 Locustana pardalina lifecycle

*L. pardalina* outbreaks are cyclic, with high-frequency events occurring in the semiarid Karoo regions, where adults gather at oviposition sites spanning up to 100 ha, to deposit their egg pods in shrubs (5, 16). Outbreaks are mainly triggered by a series of favourable seasons that allow several generations to build up, resulting in population densities capable of forming swarms (5). For oviposition, all locust species need moist soil; however, different levels of moisture at different times are necessary for the development and hatching of their eggs. For example, for desert locusts (*Schistocerca gregaria*) to successfully reproduce, at least 25 mm of rain must fall after the eggs are laid (17). This also applies to the Southern African brown locust, *Locustana pardalina* (6). This pest undergoes several developmental stages, including egg, nymph, and adult phases, with outbreaks influenced by climatic conditions (15, 18). The swarming population of brown locusts often survives until the next dry season.

**Figure 2:**
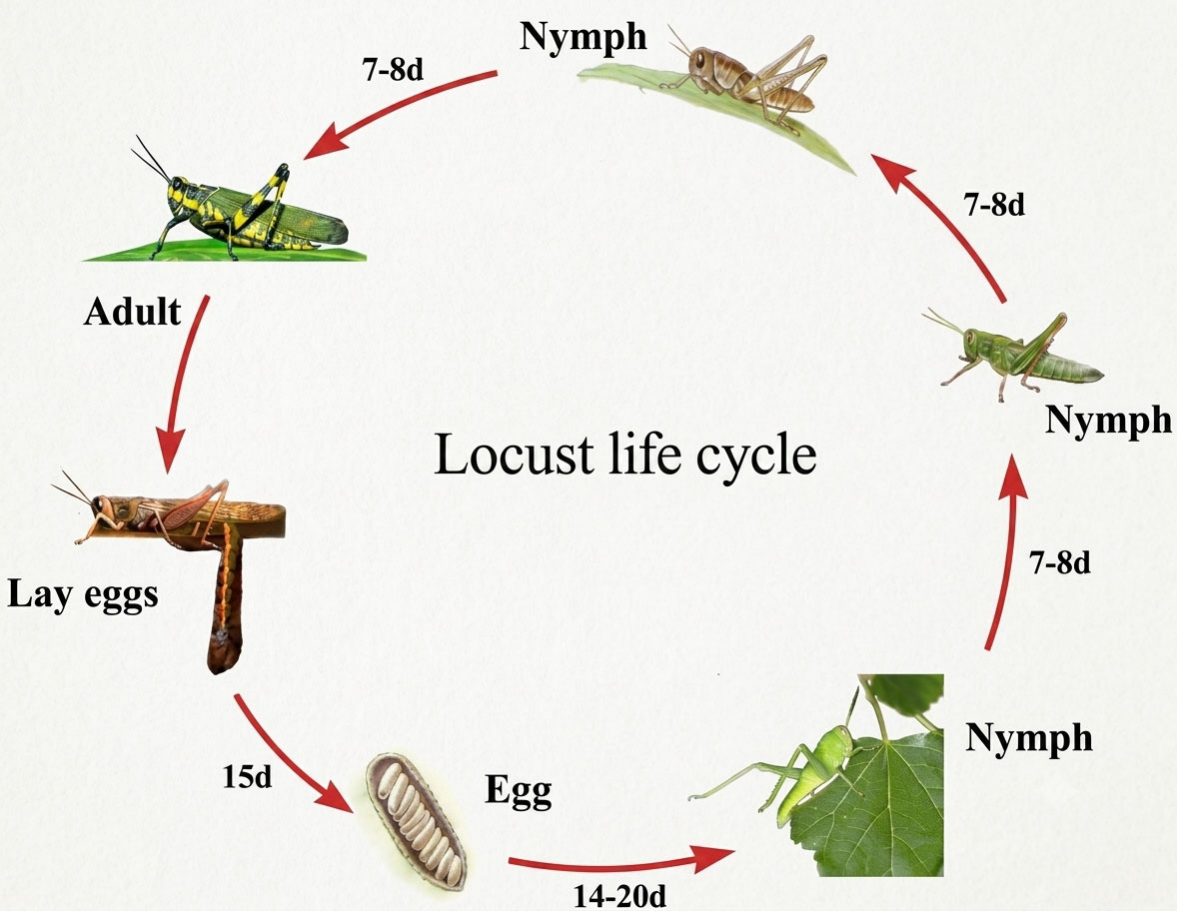
Locust developmental stages (19).

Using the tip of their abdomen, female locusts dig holes in the soil to deposit their eggs. The eggs are typically laid in clusters within pods and remain in the soil for 10 to 14 days before hatching into nymphs under favourable conditions, particularly following summer rainfall, and form a swarming population that can survive until the next dry season (12, 14), as illustrated in **Figure 2**. The incubation period can be as short as 10 to 20 days, depending on soil moisture availability and temperature. Temperatures need to exceed 8.5 °C for eggs to develop (12). Eggs develop into nymphs, or hoppers, which undergo up to five moults before reaching maturity, with each stage lasting several days to weeks (7). The nymphs, having no wings, remain on the ground and move by hopping around after emerging from their eggs (16). It is at this stage that the locusts begin feeding on vegetation for further development. The wing buds mature into full wings as they reach the adult stage after the fifth instar (16). Upon completion of the fifth moult stage, locusts become fledglings that are unable to fly due to the softness of their wings, which then take about a week to strengthen and become capable of flying (6). After several weeks and optimum wing development, the locusts can form swarms that can travel long distances, become gregarious, and voraciously feed on crops by stripping foliage (2, 7, 17).

### 3.2 The effect of *L. pardalina* infestation of economical important food crops

*Locustana pardalina* (brown locust) is considered one of the major destructive agricultural pests in Southern Africa, particularly in South Africa’s semiarid Karoo and grain-producing regions, where outbreaks are frequent (2). However, outbreaks in these regions occur approximately once every 10 years (7). When these outbreak periods occur, locust populations can increase rapidly, forming dense hopper bands and adult swarms that regularly cause extensive crop damage and damage to natural vegetation (2, 4).

Grasses (rangeland and pastures) and cereals such as *Zea mays* (maize), *Sorghum bicolor* (sorghum), *Hordeum vulgare* (barley), and *Triticum aestivum* (wheat), which this locust has a high affinity for, are the most vulnerable (2, 7, 18, 20). Both nymphs and adults feed voraciously on leaves, stems, and developing grain heads (6). This intense feeding often leads to significant leaf loss, with entire plants stripped of their foliage (7). In addition to the defoliation, stems may suffer severe injury, weakening the plant’s overall structure (4). Young seedlings are highly vulnerable and can be completely destroyed. Such damage not only causes considerable reductions in crop yield but may also culminate in complete crop failure, threatening the entire harvest. Lubinga et al. (1) reviewed that the Food and Agriculture Organization (FAO) (21) and Zhang et al. (22) reported that locust outbreaks induced cereal production losses ranging from approximately 80% to 100%. They also reported that for animal pastures, locust infestation caused production losses of approximately 80% (13).

Although legumes such as beans (*Phaseolus vulgare*), peas (*Pisum sativum*), and groundnuts (*Arachis hypogaea*), vegetables (cabbage and tomatoes), as well as oilseed crops like sunflowers (*Helianthus annus*), are not the primary target crops of this locust, they also face significant damage (6, 23). Locusts feed on these crops when their primary food supply is depleted, or they are in the path of migration (2). Locust feeding in these crops can cause substantial damage to the foliage and apical meristems (6). This damage severely impairs the photosynthetic capacity, which is responsible for growth and energy production. Overall, this impairment reduces plant vigour and yield potential (7). Consequently, both quantity and quality of harvested produce are affected, posing significant challenges to commercial production and subsistence farming. *L pardalina* outbreaks can lead to losses in expected leguminous crops in Burkina Faso, Mauritania, Namibia, and Mali, ranging from 85% to 90% (1).

While severe brown locust outbreaks have a significant impact on agricultural productivity, they also have accompanying economic consequences (24). In South Africa, the government committed more than R80 million to the locust control programme of the outbreak of 2021/22. This funding was used to purchase pesticides, spraying pumps, protective clothing, hire labourers, and rent aerial spraying services (25). During an outbreak reported in 1985–1986, the management effort involved over 250,000 hopper bands and 40,000 adult swarms in South Africa. The economic strain of locust outbreaks is often severe for farmers, who frequently have to purchase food supplies and livestock fodder or sell their livestock. In some cases, they are compelled to rely on government aid programmes to sustain their livelihoods and mitigate the detrimental effects of these infestations (2). These factors not only affect farmers but also pose broader implications for food security and market stability in the affected areas. During an outbreak in Southern Africa in the 2010s, farmers reported yield reductions ranging from 70% to 80%, with certain areas experiencing near-total losses of up to 100% (1). Such losses not only cause immediate food shortages but also significantly alter the nutrient composition of affected crops (25). As locusts consume foliage, plants are forced to reallocate resources from nutrient accumulation to survival, resulting in decreased levels of both macronutrients and micronutrients (26, 27). Research on maize and wheat indicates that the presence of locusts decreases protein, carbohydrate, and mineral levels, especially when infestations are severe (28). For instance, the protein content in maize can drop significantly by 15%–20% in infested fields. Furthermore, essential minerals like iron and zinc are significantly lower, heightening the risk of nutrient deficiencies in communities that rely on these crops for their dietary needs (28). Infested plants often display impaired root and leaf systems, limiting their ability to uptake nutrients effectively. This directly impacts food quality for both human and animal consumers. Moreover, nitrogen levels in damaged legumes, a crucial protein source, are significantly reduced (29). Consuming these crops with reduced nutrients can lead to serious health issues such as malnutrition and increased susceptibility to infections, as emphasised by the FAO in 2020 (30).

## 4 Documented outbreaks of *L. pardalina*

Outbreaks of *Locustana pardalina*, commonly known as the brown locust, have been considerably documented in Southern Africa, particularly in the semiarid regions of the Southern Nama Karoo, where the species is endemic (2). Historical and contemporary records show that brown locust outbreaks are recurrent, with population eruptions coinciding with a combination of ecological, climatic, and land-use factors (**Table 1**) (5, 6, 31). These conditions promote vegetation growth, thereby supporting rapid hopper development and population expansion (7).

**Table 1:** Factors contributing to *L. pardalina* outbreaks.

Factor	Description
Climate variability	Erratic rainfall and prolonged drought create ideal breeding conditions.
High reproductive rate	Rapid population growth occurs during wet and mild conditions.
Flight stage adaptability	Flying locusts spread rapidly and evade control.
Regional vulnerabilities	Semiarid regions like the Nama Karoo are most affected.
Control complexity	Outbreaks occur every 7–11 years despite intensive management efforts.

To ensure that the documentation of brown locust outbreaks is consistent, a combination of field surveys, farmer reports, aerial surveillance, and government monitoring programs has been carried out (6). National control agencies have established and maintained long−term records of outbreak frequency, geographic spread, swarm density, and affected agricultural areas (2, 7). Such record-keeping has been critical in identifying outbreak hotspots, particularly in the Karoo and surrounding regions, where repeated infestations have occurred over several decades. This thorough documentation not only aids in the assessment of past trends but also informs future locust management and control strategies (1).

Recent outbreak documentation has increasingly incorporated geospatial mapping and remote sensing technologies to track swarm movement and assess vegetation loss (31–33). These tools have enhanced early warning capabilities and enabled more targeted control interventions (32). Reports from major outbreak events, including those recorded in 2010, highlight the rapid increase of infestations from localised hopper bands to widespread adult swarms, often resulting in severe crop and pasture losses within short timeframes (7).

Beyond agricultural impact assessments, outbreak recording has also captured socioeconomic consequences, particularly for smallholder and subsistence farmers (1). Reports from government and extension services frequently note growing dependence on emergency control measures and food aid during severe outbreaks (2, 32). This emphasises the broader implications of the population of *L. pardalina* surges extending beyond immediate crop damage (34).

Overall, the documentation of *L. pardalina* outbreaks offers essential insights into the species’ outbreak dynamics, environmental drivers, and impacts on agricultural systems. These records serve as a basis for improving forecasting models, integrated pest management (IPM) strategies, and influencing policy responses aimed at mitigating the long−term impacts of brown locust infestations (35).

Research indicates that global yield losses may escalate by 10%–25% per additional degree of warming, highlighting the potential severity for subsistence farmers in southern Africa grappling with climatic and economic challenges. While the desert locust can migrate across continents, the brown locust tends to have a more regional impact. The brown locust also has a high reproduction rate, particularly during favourable conditions with consistent rain and mild temperatures, leading to rapid population surges capable of devastating crops across large areas (36). The rapid transition of locusts from the hopper to the flying stage poses challenges for containment efforts, as the flying stage enables them to cover larger areas and evade control measures more effectively (2). While aerial spraying efforts can manage the situation, infestations that reach irrigated crop zones, such as those along the Free State border, could cause catastrophic crop losses (36). The economic impact of these infestations can be felt for years as farmers struggle to recover from the losses incurred. These infestations directly affect the food supply chain by diminishing the availability of essential crops such as maize and lucerne, which are crucial for human consumption and livestock feed (35). Control strategies for both pests often involve early detection through satellite monitoring to track movements and behaviour patterns, followed by immediate response with chemical or biological control agents to mitigate the spread and impact of infestations. In both cases, climate change is a driving factor that intensifies the unpredictability and scale of infestations, complicating management efforts. The severity of brown locust infestations in South Africa is emphasised by substantial government investments in control and prevention efforts. During the 1985–1986 outbreak, South Africa spent over R50 million (equivalent to US$25 million at the time) and allocated an additional R14 million (around US$3.5 million) during the 1995–1996 outbreak. Despite these investments, outbreaks continue to recur approximately every 7 to 11 years, posing persistent threats to agriculture. This consistent pattern of outbreaks, intensified by climate factors, may paradoxically heighten cumulative agricultural impacts, as the pest’s population remains a continuous challenge for crop and grazing land management (34, 35). Without effective control, the damage forces farmers to depend heavily on costly feed alternatives, which increases production costs and tightens profit margins (36). This ultimately threatens the sustainability of the agricultural industry. These infestations create a cyclical impact by driving up food prices and exacerbating poverty, which is already prevalent in many rural African communities. This can lead to food insecurity and malnutrition, further perpetuating the cycle of poverty and hunger. The Food and Agriculture Organization of the United Nations’ early warning systems offer a useful framework that could be modified to handle potential brown locust outbreaks, even though the majority of locust monitoring efforts currently concentrate on the desert locust (37, 38). The danger of locust outbreaks persists despite improvements in pest management techniques. Adaptive management techniques and ongoing research are required to lessen the impact of upcoming locust invasions on agriculture. Under these circumstances, locust outbreaks/infestations pose a threat to achieving the desired yield, as evidenced by the recent FAO report that the 2020–2021 outbreak was “The deadliest in 70 years, which destroyed 70,000 ha of land in Ethiopia, Somalia, and South Asia” (4).

## 5 Current control strategies for *L. pardalina* (brown locust)

According to the study by Henschel et al. (6), the traditional approach to controlling swarms of brown locusts is reflected in South African policy, specifically the Act 11 of 1911 of the Union of South Africa.

### 5.1 Ground-based chemical control techniques

In South Africa, the control of *L. pardalina*, commonly known as the brown locust, continues to rely heavily on ground-based chemical application, especially during active outbreak phases. These interventions primarily target dense hopper bands during their early developmental stages, where localised treatment is most effective. The use of broad-spectrum insecticides, particularly synthetic pyrethroids such as deltamethrin, esfenvalerate, and alpha-cypermethrin, has remained central due to their rapid knockdown effects on *L. pardalina* populations (39, 40). In recent seasons, fipronil has been integrated into management programs for its systemic activity and residual control against multiple life stages of *L. pardalina*. Field trials have validated its efficacy, leading to formal registration for locust-specific use in South African rangelands. These insecticides are typically applied using vehicle-mounted low-volume sprayers and motorised knapsacks (e.g., Solo Port 423^®^), at calibrated dosages (e.g., 2.5 L ha^−1^), to maximise efficacy while minimising environmental drift (41). However, the continued reliance on chemical methods raises ecotoxicological concerns, particularly in ecologically sensitive zones where brown locust outbreaks frequently occur. Widespread use of these compounds has been linked to nontarget species mortality, groundwater contamination, and pesticide resistance development, necessitating a shift towards more integrated and sustainable alternatives (42, 43).

### 5.2 Biological control

Given the limitations of synthetic pesticides, there is a growing emphasis on biological control strategies specifically targeting *L. pardalina*. The entomopathogenic fungus *Metarhizium acridum*, commercially formulated as Green Muscle^®^, has emerged as a leading biopesticide for brown locust control. This strain demonstrates high host specificity towards acridid species such as *L. pardalina*, causing mortality through direct contact, infection, and proliferation within the insect body (44). In field trials conducted in semi-arid South African regions, *M. acridum* showed significant reductions in brown locust hopper density, particularly when applied during early instar stages under optimal humidity and temperature conditions. While slower-acting than chemical insecticides requiring several days to exert full lethal effects, the fungus provides the key benefit of environmental persistence and residual activity, allowing longer-term population suppression (45, 46). The integration of *M. acridum* into IPM frameworks has been advocated for *L. pardalina* due to its ecological compatibility and minimal impact on beneficial fauna. Other promising avenues for biologically managing *L. pardalina* include microbial agents, locust-specific predators, and toxic botanical compounds. For instance, Peng et al. (47) report high control success (up to 90%) using diverse biological tools, although these require habitat-specific deployment and may not be scalable for widespread *pardalina* infestations. Timely intervention against *L. pardalina* outbreaks depends heavily on early warning systems (48, 49). These systems could enhance outbreak response capability, especially in remote and rural areas where *L. pardalina* breeding sites are most common.

### 5.3 Cultural and mechanical methods

Although large-scale mechanical control is no longer widely used, traditional techniques such as ploughing egg beds, digging trenches, and manual hopper collection still offer localised benefits in the context of *L. pardalina* management. These methods, as shown in **Table 2**, may be particularly useful in resource-constrained communities or as part of integrated approaches involving school or community engagement in pest control (40). Despite advancements in IPM, significant gaps remain in the integration of ecological monitoring, predictive modelling, and socioeconomic factors. Many IPM strategies still overly depend on chemical controls, undermining long-term sustainability. Furthermore, limited adoption of biological alternatives and insufficient farmer training hinder the effective implementation of holistic IPM frameworks.

**Table 2:** Control measures used against *L. pardalina*.

Type of control	Methods/examples	Effectiveness and challenges
Traditional	Soap, paraffin oil, cattle dips, burning grass	Largely outdated, minimal efficacy
Biological	Birds, fungi, mites, forestation	Insects often die after ~ 10 days
Synthetic chemical	Organophosphates, pyrethroids	Effective but environmentally harmful
Botanical	Pyrethrum, neem, rotenone, nicotine-based products	Ecofriendly alternatives are gaining research attention
Early warning	Satellite tracking, FAO systems	Essential for timely intervention

## 6 Limitations of current locust management strategies

The effective mitigation of locust outbreaks remains a salient challenge for ensuring both food security and environmental integrity. Current intervention strategies are predominantly reactive in nature and exhibit a significant reliance on synthetic insecticidal applications, which, while offering short-term efficacy, are associated with substantial ecological and human health implications (50, 51). The inherent limitations of prevailing approaches, ranging from delayed intervention protocols to environmental conditions and a deficiency in systemic integration, underline the exigent need for more anticipatory, ecologically congruent, and sustainable methodologies. A principal deficit in current locust control strategies lies in their predilection for crisis response rather than proactive preventive measures. Many affected nations implement control interventions only subsequent to locust populations reaching economically damaging thresholds, at which juncture containment efforts become increasingly complex and resource-intensive. This reactive paradigm, as documented by El Bashir (52), engenders temporal lags in control implementation, frequently permitting swarm maturation and unconstrained dispersal. Despite their efficacy, these chemicals are broad-spectrum, impacting nontarget organisms and leading to environmental contamination. Moreover, their intensive use increases the likelihood of pesticide resistance within locust populations (42, 53–59). The harmful effects of chemicals are further discussed by Nasike (56), who investigated the impacts of synthetic insecticides used to manage desert locusts in a laboratory experiment conducted in Kenya. According to the study, bees, aquatic life, and humans are all affected by fenitrothion poisoning.

Consequently, authorities often resort to the expedited deployment of broad-spectrum pesticides, which, while providing immediate population suppression, fail to address the underlying ecological drivers of infestations or mitigate the propensity for future outbreaks (60). However, overreliance on synthetic chemical control imposes significant financial burdens and, due to global warming, creates conditions for *L. pardalina* to evolve and develop resistance (2, 6).

The extensive application of chemical insecticides encompassing organophosphate and pyrethroid compounds has elicited considerable concerns regarding their nontarget effects on biodiversity, residual persistence within ecosystems, and potential for biomagnification. While these chemical agents facilitate rapid locust mortality, they contribute to protracted ecological perturbations, including declines in pollinator populations, disruption of trophic interactions, and contamination of aquatic resources (61). In addition, this technique indirectly weakens biological control mechanisms by eliminating key natural enemies of *L. pardalina* (e.g., parasitoid wasps, predatory beetles) and disrupts essential microorganisms such as fungi like *Metarhizium*.

Furthermore, human exposure to pesticide residues, whether via spray drift, occupational contact, or contaminated food sources, presents significant public health risks, particularly within rural agricultural communities where access to and utilisation of protective measures are often constrained. A further critical limitation resides in the absence of comprehensive, integrated strategies that synergistically incorporate biological, technological, and socioecological components. Current locust control activities frequently overlook potentially efficacious tools such as habitat manipulation, conservation of natural predator populations, pheromone-based monitoring systems, and early warning infrastructures. The dynamic and migratory ethology of locusts necessitates a flexible, adaptive management framework; however, extant strategies are often characterised by rigidity, fragmentation across sectors, and dependence on contingency funding mechanisms (55). Azeem et al. (59) advocate for the adoption of IPM frameworks that actively engage local communities, implement routine surveillance protocols, and prioritise the utilisation of environmentally benign biocontrol agents to achieve long-term, sustainable outcomes. Numerous locust-affected regions encounter significant logistical impediments, including inadequate infrastructural capacity, limited specialised training for field personnel, suboptimal inter-agency coordination, and inconsistent data dissemination, as demonstrated by Baraka et al. (62) in Kenya.

The major challenges of current brown locust management strategies are ecological damage (synthetic insecticides), high financial costs, and, most critically, the locust’s ability to develop resistance, resilience, and adaptive evasion across its lifecycle.

South Africa’s rich botanical biodiversity offers opportunities to overcome limitations via ethnobotanical solutions, which include traditional plant-based knowledge used for centuries in healthcare and pest management. Bioinsecticides from indigenous plant extracts contain properties to combat resistant and evolving brown locust populations while reducing human and environmental harm as a sustainable alternative. This approach aligns with sustainable pest management by leveraging natural plant compounds that may disrupt *L. pardalina* development without the ecological collateral damage of synthetic chemicals.

According to Isman (63), botanical insecticides have gained significant scholarly attention over the past two decades. Rich in diverse secondary metabolites, plant extracts demonstrate considerable potential as eco-friendly pest control agents, functioning as repellents, insecticides, or insect growth disruptors. Moreover, research further suggests that a focus on plant properties and the combination of plant-derived compounds can enhance their efficacy through synergistic effects, offering a viable substitute for synthetic insecticides (64, 65). This approach has been widely explored in pest management, underscoring its effectiveness in mitigating resistance. Plant-based solutions offer a sustainable alternative by targeting pests with diverse bioactive compounds, thereby reducing reliance on synthetic pesticides. Their biodegradability and lower toxicity to nontarget species address environmental and health concerns. Additionally, their adaptability within IPM frameworks enhances resilience against pesticide resistance.

## 7 The potential of botanical insecticides for *L. pardalina* control

The control of *Locusta pardalina* (Walker) remains persistently challenging for agricultural communities (7). Control efforts have ranged from rudimentary measures such as spraying hopper band soap solution, paraffin oil, cattle dip formulation, and burning infested grassland (6), to the adoption of innovative biological and synthetic methods (1, 2, 47, 66). These methods were either ineffective or harmful to humans, animals, nontarget insects, and the environment (67, 68). This has compelled researchers to investigate alternative methods that prioritise ecological balance while effectively managing pest populations (1).

In search of safe and ecologically balanced pest control methods, botanical insecticides have emerged as promising alternatives to chemical pesticides in managing agricultural pests, including *L. pardalina* (2). Botanical insecticides are defined as natural pesticides extracted or derived from plants. They are also referred to as natural insecticides (63). These natural extracts contain bioactive compounds that can disrupt pest behaviour, reproduction, or survival (17). Plant extracts offer several advantages over synthetic chemicals. They are biodegradable, which means they break down naturally in the environment (69). This characteristic reduces the risk of environmental contamination (70). Additionally, plant extracts are generally safer for nontarget species, such as beneficial insects and humans, minimising potential harm (71, 72). Moreover, plant extracts reduce the chances of pests developing resistance (70). Several plant species with insecticidal properties effective against locust and grasshoppers have been identified. The botanical pesticides that have been identified include nicotine, rotenone, sabadilla, ryania, rutin, pyrethrum, oleuropein, plant essential oils, pyrethrin, and azadirachtin. The plants from which these pesticides have been extracted include neem (*Azadirachta indica*), Vogel’s tephrosia (*Tephrosia vogelii*), African marigold (*Tagetes erecta*), lemon bush (*Lippia javanica*), *Capsicum annum*, and *Chryanthemum* or *Tenacetum* spp. Most used botanical products include pyrethrum, rotenone, neem, and essential oils (72). Others include ryania, nicotine, sabadilla, garlic oil, and capsicum oleoresin, which are used regionally in low volumes (70).

### 7.1 Pyrethrum

Pyrethrum is a botanical insecticide derived from the flower heads of the perennial plant *Tanacetum cinerariifolium* (or *Chrysanthemum cinerariifolium*) of the family Asteraceae, commonly known as the Dalmatian chrysanthemum (73). This plant is characterised by yellow flowers and feathery green leaves that make it an appealing, noteworthy species in gardens and ecological studies. The flower heads of this plant are dried and pulverised to extract insecticidal ingredients, including pyrethrum (73, 74). The name pyrethrum is also used for the crude extract of these plants (75). Insect mortality is associated with the insecticidal properties of pyrethrum. Since then, pyrethrum has undergone extensive research aimed at establishing its complete, effective, and safe commercial exploitation as a source of natural insecticides collectively known as pyrethrins (75). Its insecticidal activity is attributed to a group of six esters collectively known as pyrethrins, which have been widely used for the control of agricultural pests, including orthopterans (76). The *“*pyrethrins*”* are meroterpenes (mixed biosynthesis of a terpene-derived unit attached to a nonterpene moiety), esters of chrysanthemic or pyrethric acid with keto cyclopentene alcohols: pyrethrolone (pyrethrins I and II), cinerolone (cinerins I and II), and jasmolone (jasmolin I and II) (68). It is often available at 25%–50% concentration, with pyrethins I and II present in greater amounts (75).

*Pyrethrum* is effective when sprayed directly on active pest insects that are visible. In insects, it acts primarily on the nervous system by disrupting voltage−gated sodium channels, prolonging channel opening, and causing repetitive nerve firing (77). This leads to hyperexcitation, paralysis, and rapid knockdown of insects following contact exposure. Locusts and grasshoppers, which possess high neuromuscular activity and metabolic demand, are particularly susceptible to this mode of action, resulting in rapid hyperactivity, immobilisation, and mortality (77). Field and laboratory studies show that pyrethrum has strong contact toxicity against both nymphal and adult stages of grasshoppers and locusts (77). Pyrethrum formulations have been shown to reduce feeding activity rapidly, thereby limiting immediate crop damage during outbreaks. Its fast knockdown effect makes it particularly useful for localised control of hopper bands, where immediate suppression is required to prevent defoliation (73, 77). In Kenya, farmers were encouraged to use pyrethrum-producing flowers from a local plant to combat locust swarms while waiting for aid from FAO and government assistance (62). One of the significant advantages of using pyrethrum in the management of locust and grasshopper is its rapid environmental degradation (75). Pyrethrins are regarded as an environmentally friendly biopesticide due to their quick degradation when exposed to sunlight and air exposure, resulting in low persistence in soil and minimal long−term residue accumulation (77). This characteristic aligns well with IPM and organic farming systems, especially in smallholder farming systems that prioritise environmental safety. In addition, natural pyrethrins are moderately toxic to mammalian organisms, with oral acute LD_50_ values in rats ranging from 350 to 500 mg kg^−1^ when applied according to recommended guidelines, whereas technical-grade pyrethrum is less toxic (~ 1,500 mg kg^−1^) (75). Despite these benefits, pyrethrum negatively affects nontarget organisms, including pollinators (bees) and natural enemies of pests (aquatic organisms). This necessitates careful timing and localised application strategies to minimise ecological impact. Furthermore, the absence of residual activity limits long−term protection, requiring repeated applications during prolonged locust outbreaks. Pyrethrum is one of the few insecticides allowed for use in certified organic production of crops in the USA, Europe, Australia, and New Zealand. The synergists piperonyl butoxide (PBO) and MGK-264, however, are not certified for use on organic crops, so growers who aim to meet organic certification standards need to be aware of this.

### 7.2 Rotenone

*Rotenone* is another botanical insecticide that has been used to manage locusts, grasshoppers, and other pests in both cultivated fields and livestock (72). It is a colourless, odourless, crystalline isoflavone derived from the roots, leaves, seeds, and bark of *Tephrosia vogelii*, belonging to the family Leguminosae (73). It is native to tropical regions of Africa and can grow to a height of 2 to 3 m in a growing season of 5 to 7 months (78). It produces flowers that are white, purple, or red (79). It produces various compounds such as flavonoids, steroids, and rotenone (80). Its formulation includes dust, crystalline preparations, and emulsifiable solution (78). As dust, it is used with other pesticides such as carbaryl, pyrethrins, piperonyl butoxide, lindane, and others in pesticide products to control a variety of insect pests and undesirable fish (79–81). Rotenone causes neurotoxicity by disrupting cellular respiration. It does this by inhibiting the mitochondrial electron transport chain, specifically blocking the transfer of electrons from iron to sulphur centres in complex I to ubiquinone, thus affecting ATP synthesis (79, 81–84). It also generates mitochondrial reactive oxygen species (ROS), which induce apoptosis (82, 84). In insects, rotenone functions through contact and stomach action, exhibiting secondary acaricidal properties (78), and induces necrosis, mitochondrial dysfunction, cytotoxic effects, and cytoplasmic membrane damage (83). Al-Al-Maroug et al. (74) demonstrated that rotenone exerted potent toxic effects and induced antifeedant behaviour in desert locust, *Schistocerca gregaria* nymphs. These studies highlight the significant effects of rotenone on the survival and feeding behaviour of locust pests, suggesting that *Tephrosia* containing rotenone could potentially offer a promising new avenue for pest management and mitigation strategies. Although the use of rotenone is permitted in organic farming when pest infestations are significantly high, it is restricted in some countries due to toxicity to invertebrates, humans, and pigs. Research does, however, show that insecticides consisting of rotenone are not toxic to bees unless combined with pyrethrum (78). Countries that permit the use of retonone as an insecticide include Denmark, the Netherlands, Portugal, the USA, and Slovenia. In South Africa, rotenone is used to control invasive fish species (74).

### 7.3 Neem

Neem is a fast-growing tropical tree with medicinal and insecticidal properties. Its scientific name is *Azadirachta indica* (a member of the mahogany family, Meliaceae) (71). This species primarily originated in southern and southeastern Asia. Today, *A. indica* has adapted and spread across tropical and subtropical regions, specifically parts of Africa, the USA, and Australia. Twenty years later, neem was introduced to other countries primarily for afforestation and fuelwood production in dry areas, as well as for additional purposes, including its use as an avenue or shade tree and as a source of natural pesticides (85). The insecticidal properties of this tree can be effectively obtained from the leaves and seed kernels (86). As an insecticide, neem is used in various formulations, including powders, seed extracts, or emulsifiable oils (86). Researchers have revealed that neem contains several insecticidal ingredients, including azadirachtin, salanine, limonoid, meliantriol, and nimbin. These compounds are tetranotriterpenoids that occur in most parts of the tree (74). Among these chemical compounds, azadirachtin has garnered significant attention as the principal biocidal active ingredient for anthropods, including *Locusta pardalina* (83). It affects a variety of metabolic processes, including feeding behaviour, oviposition deterrence, metamorphosis inhibition, hormonal regulation, protein synthesis, changes in biological fitness, and chitin synthesis (70). It also interferes with moulting and hampers reproductive cycles, ultimately leading to population decline (71). A study conducted in the laboratory, semifield, and field conditions in Africa, Germany, and Madagascar revealed that neem oil had a very strong phagorepellent effect on the desert locust, Schistocerca gregaria, the red locust, *Nomadacris septemfasciata*, and the variegated grasshopper, *Zonocerus variegatus*, and a lesser repellent effect on the African migratory locust, *Locusta migratoria migratorioides* (87). In another study, Abdelbagi and colleagues (88) showed that treatment of desert locust nymphs with neem seed powder (NSP) exhibited a superior systemic effect compared to other forms of neem seed products, as it prevented further development, deterred feeding, and confined the locusts to the breeding sites. For neem extracts to cause these effects on *L. pardalina*, they must be in direct contact with the insect or ingested (70). Neem has been shown to have low toxicity to nontarget organisms, including humans and beneficial insects, and biodegradability in the environment, making it an ecofriendly alternative to synthetic pesticides (71). Although research on *L. pardalina* may be limited, the general effectiveness of neem*’*s azadirachitin on several locust species suggests it would likely be effective in mitigating the damage caused by *L. pardalina* (89–92). Through this research, neem extracts had promising results in mitigating the damage caused by *Locusta pardalina*. However, the efficacy of neem varies with environmental factors such as temperature, humidity, and the developmental stage of the locusts. Future studies are needed to optimise neem extract application rates and timing to maximise its effectiveness in the field.

### 7.4 Tobacco plant

The other botanical insecticide that has been used in the control of locust species is *Nicotiana sylvestris* (93). The role of *N. silvestris* as a botanical insecticide is attributed to the presence of tannins, saponins, flavonoids, alkaloids, phenols, and glycosides. These compounds have been isolated in other plants and have played an important role in protecting against plant-feeding insects and herbivores. The primary alkaloid in *N. silvestris* is nicotine (92, 94). Neonicotinoid, a synthetic derivative of nicotine, has also gained popularity in the agricultural sector due to its effectiveness in controlling a wide range of agricultural pests, including locusts (94–96). Its use as an insecticide stemmed from its remarkable pharmacological properties and alleged health hazards associated with tobacco smoking (97). Neonicotinoids target the central nervous system of insects by binding to nicotinic acetylcholine receptors, causing overstimulation and eventually leading to paralysis and death (98, 99).

In *Locusta pardalina* control, neonicotinoids such as imidacloprid and thiamethoxam have shown substantial efficacy (71). These compounds effectively hinder nerve transmission in locusts, resulting in impaired movement and significant feeding difficulties, which can ultimately lead to their demise (100). The rapid action of neonicotinoids makes them ideal for controlling locust outbreaks, where swift population control is critical to avoid significant crop damage (100).

Neonicotinoids operate in a systemic mode of action, meaning that when applied to plants, they effectively travel throughout the plant tissues, and every part of the plant is protected from locusts ingesting it (99). Moreover, the relatively low application rates required for neonicotinoids make them cost-effective in large-scale pest control operations (82). However, despite their efficacy, the widespread use of neonicotinoids has undeniably raised environmental concerns, especially regarding their detrimental impact on nontarget species, such as pollinators and aquatic organisms (98, 99). Extracts from *Ruta chalepensis*, *Rhazya stricta*, *Heliotropium bacciferum*, *Salvadora persica*, and *Moringa olcifera* have been shown to repel *Oryzaephilus suranimensis* (101), while extracts from *Lantana camara*, *Ricinus communis*, *Gliciridia sepium*, *Calotropis giganta*, and *Carica papaya* have been shown to successfully suppress *Sitophilus oryzae* activity on food grains (102). This makes medicinal plant extracts a highly effective option for integrated pest management programs aimed at combating locust damage while promoting environmental sustainability (43). The most used botanical products include pyrethrum, rotenone, neem, and essential oils, as shown in **Table 3**.

**Table 3:** Comparison of medicinal plant-based insecticides for *L. pardalina* control.

Botanical extract	Source plant(s)	Active compounds	Mode of action	Advantages	Limitations
Pyrethrum	*Tanacetum cinerariifolium* (Asteraceae)	Pyrethrins (I, II, etc.)	Neurotoxic—affects sodium channels	Fast-acting, allowed in organic farms	Light-sensitive, needs direct contact
Rotenone	*Tephrosia vogelii*	Rotenone, deguelin	Mitochondrial disruption, ROS-induced apoptosis	Broad spectrum	Toxic to fish/humans in high doses
Neem	*Azadirachta indica*	Azadirachtin, nimbin, salanine	Interferes with moulting, reproduction, and protein synthesis	Biodegradable, safe for nontargets	Efficacy varies with the environment
Nicotinoids	*Nicotiana* silvestris	Nicotine, nornicotine, and anabasine	Targets nicotinic receptors, systemic action	Fast-acting, systemic, cost-effective	Environmental toxicity, regulatory concerns

## 8 Mechanisms of action of botanical insecticides against locusts

Employing plant-derived substances to manage locust populations presents a potentially sustainable and environmentally considerate approach compared with conventional synthetic treatments. The primary mechanisms by which these natural agents influence locusts involve feeding deterrence, antifeedant activity, toxicity, growth inhibition, and reproductive interference. These actions collectively interfere with food consumption patterns, lifecycle progression, and the ability of locusts to reproduce, consequently limiting population expansion and the likelihood of large infestations.

### 8.1 Antifeedant effects

The antifeedant works by controlling locust behaviour, effectively deterring them from feeding on plants. This helps protect crops and minimise yield losses, which is crucial for farmers and the agricultural sector (103, 104). Several plant species have demonstrated notable antifeedant properties against locusts, effectively discouraging consumption and hindering nutrient uptake. Observations indicated that *Ruta chalepensis* and *Peganum harmala* significantly decreased food intake and nutrient processing in *Schistocerca gregaria*, suggesting a strong feeding deterrent effect (105, 106). Similarly, *Azadirachta indica* (neem) showed pronounced antifeedant effects, with studies indicating slowed development and reduced feeding in younger locusts (107). Such deterrent effects could be important in reducing agricultural damage during locust outbreaks. In a 2020 study, the chemosensory protein (CSP) in *Locusta migratoria* was associated with antifeedant responses. The feeding behaviour findings revealed that the *Lmig*CSPIII locusts had reduced sensitivity to the antifeedant (108).

### 8.2 Toxicity and mortality

Numerous medicinal plants also possess inherent insecticidal properties. An extract from *Mucuna pruriens* was found to cause high mortality rates in locusts through both contact and ingestion, highlighting its potential as a natural pest control agent (106). Furthermore, oils derived from *Jatropha curcas* and *A. indica* significantly increased mortality rates and suppressed reproductive success in treated locust groups, with neem oil reducing egg hatching by 58.54% (109).

### 8.3 Synergistic effects of plant oils

**Figure 3:**
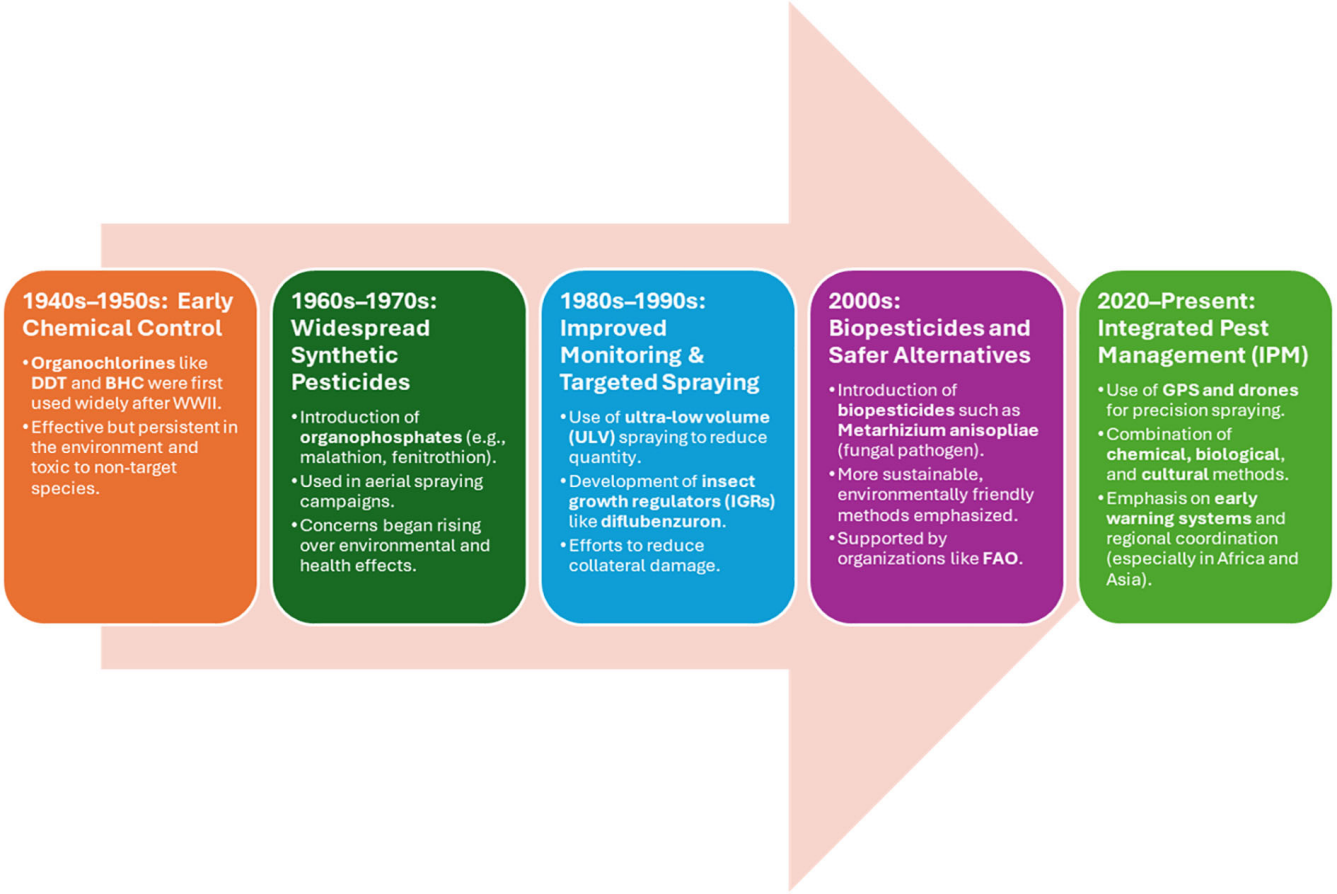
Timeline of the evolution of locust outbreak management approaches.

The effectiveness of natural insect control agents can be improved through combined applications. A specific blend of essential oils, including *Carum carvi*, *Citrus sinensis*, and *Gaultheria procumbens*, resulted in 100% mortality in *S. gregaria* within 24 h of exposure, highlighting synergistic activity of plant compounds (110). However, the success of plant-based locust management may be influenced by environmental variables such as temperature, humidity, formulation stability, and locust developmental stage.Targeting the physiological traits of *Locustana pardalina* can improve plant-based pest control efficacy. Growth and reproduction depend heavily on hormonal regulation, such as juvenile hormones and ecdysteroids, which compounds like azadirachtin disrupt, impairing molting and reproduction (21, 110). Digestive enzymes can also be inhibited by phytochemicals such as tannins, saponins, and alkaloids, reducing feeding efficiency (104, 105). Neuroactive alkaloids, notably nicotine from *Nicotiana sylvestris*, act on insect nervous systems by overstimulating nicotinic acetylcholine receptors, leading to paralysis and death, a mechanism also mimicked by synthetic neonicotinoids (21). Essential oils further enhance control by disrupting respiration and water balance through cuticular penetration (103). These combined mechanisms highlight the strong potential of botanical agents as eco-friendly alternatives for locust management (**Figure 3**).

## 9 Future research and recommendations

Despite promising findings, research specifically targeting Locustana pardalina remains limited. Laboratory bioassays are needed to evaluate *Azadirachta indica*, *Chrysanthemum cinerariifolium* (pyrethrum), *Mucuna pruriens*, and *Jatropha curcas* extracts (103). While neem shows physiological effects on *Schistocerca gregaria*, its efficacy against *L. pardalina* remains unverified. Similarly, although *M. pruriens* extracts are toxic to *L. migratoria*, species-specific effects require further testing (104). Field trials are necessary to evaluate environmental influences such as humidity, UV exposure, and temperature on botanical insecticide performance. Plant oil mixtures exhibit synergistic toxicity, highlighting the need for ecological safety assessment (105). *Metarhizium anisopliae* also shows humidity-dependent efficacy (106). Integration of botanicals with synthetic and biological control agents is recommended, as *Jatropha curcas* has demonstrated effectiveness in multitrophic systems (107). Finally, pilot field studies should assess dosage, stability, cost-effectiveness, and farmer adoption to support practical implementation in semi-arid agroecosystems (108–111).

## 10 Conclusion

Recurrent brown locust outbreaks across the globe, particularly in Africa, endanger rural livelihoods and food security. Ecofriendly pest management techniques, such as the use of botanical insecticides to control the brown locust, offer a viable and affordable substitute for traditional chemical pesticides. Thus, reducing locust populations with the help of natural bioactive compounds found in medicinal plants can minimise environmental damage and preserve biodiversity. Medicinal plants are more cost-effective than synthetic chemicals because they can be found locally and only require a small amount of processing. Their application also reduces the possibility of pest resistance while preserving the environment, human health, and nontarget organisms. The incorporation of botanical pesticides into locust control programs could revolutionise pest management as the demand for more environmentally friendly and sustainable agricultural practices increases. To optimise these plant-based strategies for large-scale implementation and ensure long-term food security and ecological sustainability in regions such as Southern Africa, ongoing research and field trials are essential.
